# The Missouri Valley Veterinary Medical Association

**Published:** 1898-01

**Authors:** 


### THE MISSOURI VALLEY VETERINARY MEDICAL ASSOCIATION.

THE annual meeting was held on December 8th in the lecture-room of the Kansas City Veterinary College. A fair number of members were in attendance. Among other matters of interest, Dr. R. H. Harrison reported a case of "Rumenotomy" and one of "Rabies." Dr. Sihler a case of "Nasal Polypi," with a novel operation for its removal by making an incision into the trachea, larynx, and pharynx. A paper on "Pericarditis in Cattle," with a number of records of cases, was presented by Dr. R. C. Moore. "Wounds and Injuries to the External Ear" was the subject of a paper by Dr. R. P. Steddom. These reports and cases excited a great deal of interest and were freely discussed by the various members.

